# High-resolution *in vivo* imaging of regenerating dendrites of *Drosophila* sensory neurons during metamorphosis: local filopodial degeneration and heterotypic dendrite–dendrite contacts

**DOI:** 10.1111/gtc.12008

**Published:** 2012-11-15

**Authors:** Daisuke Satoh, Ritsuko Suyama, Ken-ichi Kimura, Tadashi Uemura

**Affiliations:** 1 Graduate School of Biostudies, Kyoto UniversityKyoto, 606-8501, Japan; 2 Hokkaido University of EducationSapporo Campus, Sapporo, 002-8502, Japan

## Abstract

Neuronal circuits that are formed in early development are reorganized at later developmental stages to support a wide range of adult behaviors. At *Drosophila* pupal stages, one example of this reorganization is dendritic remodeling of multidendritic neurons, which is accomplished by pruning and subsequent regeneration of branches in environments quite distinct from those in larval life. Here, we used long-term *in vivo* time-lapse recordings at high spatiotemporal resolution and analyzed the dynamics of two adjacent cell types that remodel dendritic arbors, which eventually innervate the lateral plate of the adult abdomen. These neurons initially exhibited dynamic extension, withdrawal and local degeneration of filopodia that sprouted from all along the length of regenerating branches. At a midpupal stage, branches extending from the two cell types started fasciculating with each other, which prompted us to test the hypothesis that this heterotypic contact may serve as a guiding scaffold for shaping dendritic arbors. Unexpectedly, our cell ablation study gave only marginal effects on the branch length and the arbor shape. This result suggests that the arbor morphology of the adult neurons in this study can be specified mostly in the absence of the dendrite–dendrite contact.

## Introduction

The dendritic arbor of the neuron is the subcellular compartment that receives and processes synaptic or sensory inputs, thereby effecting appropriate responses of animals (Hausser & Mel 2003; London & Hausser 2005). Because its architecture influences the neuronal function, for example, by restriction of the number and type of inputs, it is important to investigate how dendrites acquire their characteristic size and morphology (Jan & Jan 2010). The spatial pattern of the dendritic arborization is not fixed by branch elongation in early development, and it is also sculpted at later stages by branch retraction and elimination, and the mature pattern is maintained throughout animal life (Wong & Ghosh 2002; Luo & O'Leary 2005).

Dendritic arborization (da) neurons constitute the largest subset of multidendritic neurons in *Drosophila*, and their stereotyped organization has provided a versatile model system, where we can investigate genetic programs controlling a number of important structural and functional aspects of dendrites (Corty *et al*. 2009; Jan & Jan 2010). These include early development and elaboration of dendritic arbors (Gao *et al*. 1999; Satoh *et al*. 2008; Zheng *et al*. 2008), the diversity of arbor morphology (Grueber *et al*. 2003; Sugimura *et al*. 2004; Hattori *et al*. 2007), processing of multimodal sensory inputs (Im & Galko 2012), lesion-induced regeneration (Sugimura *et al*. 2003; Tao & Rolls 2011; Song *et al*. 2012), degeneration (Lee *et al*. 2011; Wen *et al*. 2011) and dendritic remodeling during metamorphosis (Williams & Truman 2004, 2005b; Kuo *et al*. 2005; Lee *et al*. 2009; Kirilly *et al*. 2011).

Da neurons grow two-dimensional dendrites underneath the epidermis and on the musculature during late embryonic and larval stages (Han *et al*. 2012; Kim *et al*. 2012). During metamorphosis, larval dendritic arbors of surviving neurons are totally pruned and subsequently regenerated, and many of the rebuilt arbors persist throughout adult life (Williams & Truman 2005a; Shimono *et al*. 2009; Yasunaga *et al*. 2010). This large-scale structural conversion from larval to the adult type is considered to be one example of dendritic and axonal reorganizations without death of the parent neurons, which is crucial for supporting stage-specific behaviors of the animal (Consoulas *et al*. 2000; Luo & O'Leary 2005). It should be noted that after the pruning, regenerating neurons do not simply recapitulate dendrite formation in early development, because they are exposed to environments that are distinct in spatial and hormonal respects from those in the embryo and larva. For example, larval da neurons regulate their growth in coordination with the expanding body wall (Parrish *et al*. 2009); however, pupal da neurons somehow have to control their arbor shape and size in a body whose volume has been predetermined by the nutritional status during larval development (Hietakangas & Cohen 2009; Mirth & Shingleton 2012). Thus, investigating programs controlling the dendritic remodeling is expected to provide novel mechanistic insights into dendrite morphogenesis.

**Figure 1:**
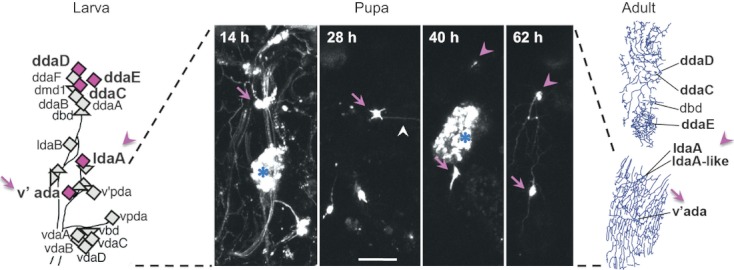
Time-lapse recording of two types of da neurons during metamorphosis: v'ada and ldaA/ldaA-like. (left) A diagram of positions of multidendritic (md) neurons in an abdominal hemi-segment (A2–A7) of the *Drosophila* larval peripheral nervous system. Diamonds represent individual da neurons, and triangles represent other types of md neurons. Magenta diamonds with bold letters highlight five da neurons that survived metamorphosis. Adapted from fig. 4A of Shimono *et al*. (2009). (middle) Selected frames of the pruning and regeneration phases that focused on the ventral region of the hemi-segment (see also Movie S1 in Supporting Information). Pupal stages are indicated as hours after puparium formation (APF). In these images, we were able to track v'ada (magenta arrow) throughout this recording, whereas ldaA/ldaA-like was first identified approximately 40 h APF (magenta arrowhead). A posterior-directed branch remained at 28 h APF (white arrowhead) and was subsequently pruned. Dissociated larval fat body cells are marked with blue asterisks (Nelliot *et al*. 2006). Genotype: *Gal4*^*109(2)80*^ *UAS-mCD8::GFP*/*Gal4*^*109(2)80*^ *UAS-mCD8::GFP*. Scale bar, 50 μm. (right) Tracings of dendrites and cell bodies in a female of 1–3 days after eclosion. Adapted from fig. 5F,J of Shimono *et al*. (2009). Anterior is to the left and dorsal is up in this and subsequent figures, unless described otherwise.

One prominent advantage of observing pupal da neurons is that they are accessible to long-term *in vivo* time-lapse recordings. In fact, regeneration and elaboration of a dorsolateral da neuron, ddaE, can be tracked for up to nearly 2 days, and this neuron actively migrates to a new position on the body wall (Williams & Truman 2004). We previously provided a systematic anatomical map of all persistent neurons (Shimono *et al*. 2009), whereby we can study intrinsic and extrinsic mechanisms of dendritic remodeling *in vivo*. In this study, we focused on three other da neurons: ldaA, ldaA-like and v'ada whose expansive arbor occupies the entire lateral plate of the abdomen (right in Fig. 1; also shown later in Fig. 3G). ldaA and ldaA-like are a pair of closely associated cells that develop arbors of similar morphology (Shimono *et al*. 2009), and we hereafter designate them as ldaA/A-like that belong to a single cell type. These two cell types, v'ada and ldaA/A-like, protruded filopodia from regenerating branches and underwent cycles of extension and retraction and local degeneration. Branches extending from the two cell types overlapped with each other, and we explored its functional relevance.

## Results and Discussion

### Dynamic extension, withdrawal, degeneration and stabilization of filopodia of regenerating branches

We tracked v'ada neurons from the large-scale pruning phase of the dendrites until their regeneration over a period of 2 days (Fig. 1 and Movie S1 in Supporting Information). After larval branches were pruned and cleared, v'ada cell bodies started shifting ventrally (arrows in Fig. 1). Subsequently what appeared on the focal planes approximately 40 h after puparium formation (APF) was ldaA/A-like with ventrally extending branches (arrowheads in Fig. 1), which encountered dorsally growing branches of v'ada. Below, we describe our detailed observations of regenerating v'ada branches and then focus on the dendrite–dendrite contact between v'ada and ldaA/A-like neurons.

**Figure 2:**
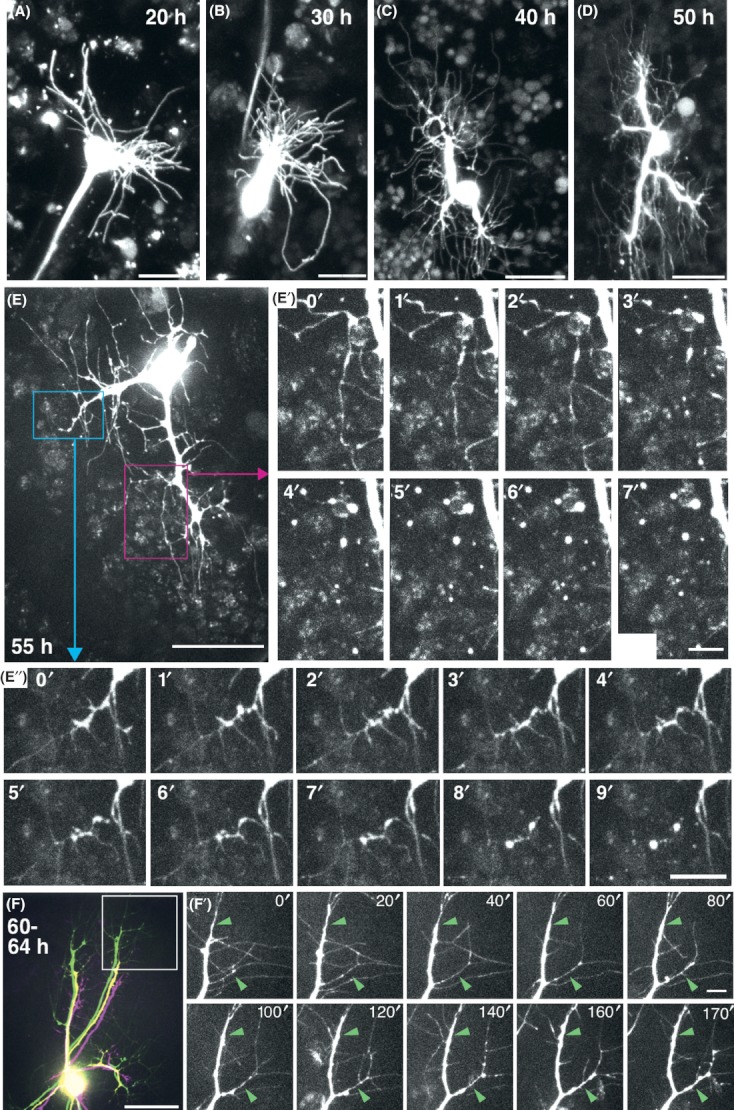
Regenerating dendritic branches of v'ada after the pruning phase during metamorphosis. (A–D) Representative images of v'ada at 20 h (A), 30 h (B), 40 h (C) and 50 h (D) after puparium formation (APF). Long filopodia protrude from the cell body (A) and also from primary branches (B–D). (C and D) Growth cone–like structures were observed at tips of the primary branches (see also Movie S2 in Supporting Information). An axon extends toward the left below in ‘A’; in ‘B’–'D', axons extending deep inside the bodies are not seen in these images. (E–E”) Local degeneration of long filopodia of v'ada at 55 h APF. (E' and E”) High-power 1-min interval images of the boxed regions in ‘E’ (see also Movie S3 in Supporting Information). (F) Growth of primary branches at 60–64 h APF. Images taken at 60 h (magenta), 62 h (yellow) and 64 h APF (green) were overlaid (also see Movie S4 in Supporting Information). (F') High-power 20-min interval images of the tip of the extending branch (white boxed region in ‘F';) at 64 h APF onwards. Green arrowheads mark fixed points throughout the recording, which shows conversions of thin filopodia into stable branches. No filopodia, except for those marked, remained stable throughout this time period. See also Movie S5 (Supporting Information). Genotype: *ppk-EGFP/ppk-EGFP*. Scale bars, 25 μm (A–E and F) and 5 μm (E', E'', and F').

To monitor dynamics of dendrite regeneration, we performed time-lapse recordings for several hours at high spatiotemporal resolution, at multiple pupal stages. As soon as larval dendrites were pruned, dynamic processes protruded from the v'ada cell body (Fig. 2A), followed by the emergence of primary branches (Fig. 2B–D). Tips of the primary branches were capped with growth cone–like structures (Movie S2 in Supporting Information). The processes longer than 20 μm were distributed along the entire length of the branches, and they underwent active extension and withdrawal. Throughout this study, we designate these dynamic processes as filopodia and distinguish them from branches that were thicker and more stable.

Until approximately 55 h APF, we occasionally observed degeneration of filopodia (Fig. 2E–E” and Movie S3 in Supporting Information). Compared to the branch pruning by 20 h APF (Williams & Truman 2005a), this degeneration was more local and rapid. Degradation was restricted to the filopodia, whereas primary branches were not severed or retracted. Detached filopodia were entirely fragmented within 10 min, in contrast to an hour that was taken by the branch pruning (e.g., see fig. 3 of Williams & Truman 2005a). This time difference may reflect distinct cytoskeletal organizations between the degenerating filopodia and the pruned branches that are rich in microtubules (Williams & Truman 2005a; Lee *et al*. 2009). The degeneration could be reminiscent of axonal degradation in the zebrafish peripheral nervous system, where repulsive interactions between isoneuronal axons occasionally execute local degradation and control shapes and sizes of sensory arbors (Sagasti *et al*. 2005; Grueber & Sagasti 2010), and also in the nociceptive neuron PVD of *Caenorhabditis elegans*, where dendrites are locally broken off after dynamic outgrowth, and it is proposed that this series of events eliminates extra branches (Oren-Suissa *et al*. 2010; Smith *et al*. 2010). It remains to be elucidated whether the degradation we found contributes to final spatial patterns of regenerating dendritic branches or not. The axonal degeneration in the fish system is triggered also by contacts between different cells; likewise, we did find instances where branches of v'ada encountered those of ldaA/A-like and then filopodia were degraded (described later in Fig. 4A and Movie S6 in Supporting Information).

At 60 h APF onwards, the arbor underwent a persistent increase in complexity and size with a rapid expansion of primary branches and generation of higher-order branches, and still displayed high filopodial activity on both tips and stalks of the branches (Fig. 2F–F', Movies S4 and S5 in Supporting Information). Filopodia at the tips were converted into thicker and stable branches; similarly, a subset of filopodia on the stalks was stabilized to become collaterals (top and bottom arrowheads, respectively, in Fig. 2F'; Movie S5 in Supporting Information).

### Branches of v'ada and ldaA/A-like neurons start fasciculating with each other at a midpupal stage

**Figure 3:**
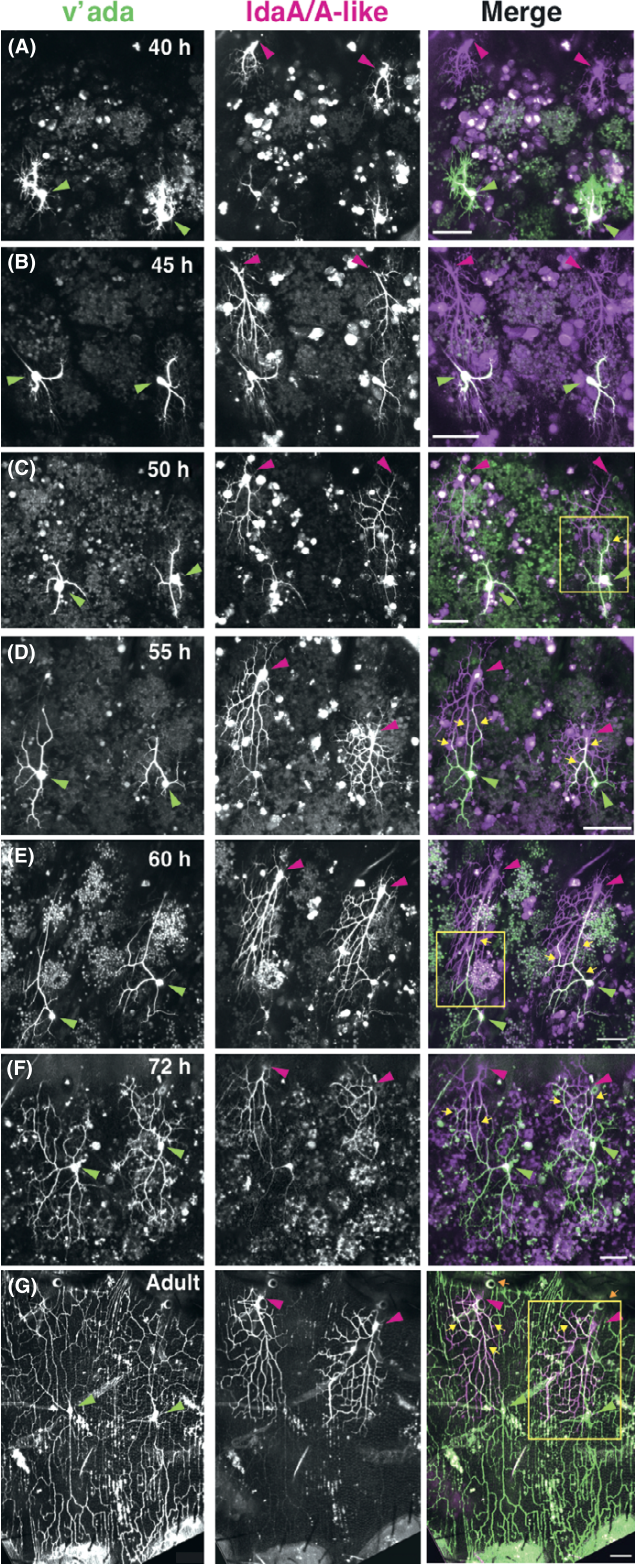
Imaging of v'ada and ldaA/A-like from pupa to adult. (A–G) v'ada (left column, green in the merged images at right) and ldaA/A-like (middle column, magenta in the merged images) at distinct pupal stages indicated (A–F) and at 2–3 days after eclosion (G). Throughout this figure, green and red arrowheads mark cell bodies of v'ada and ldaA/A-like neurons, respectively. Yellow arrows (merge in C–G) point to overlaps of v'ada and ldaA/A-like dendritic branches. Three boxed regions in ‘C’, ‘E’ and ‘G’ are enlarged in Fig. 4B–D, respectively. In the middle panels of ‘A’–'F';, note that v'ada neurons were also labeled more weakly by the driver used (*C161-Gal4*), than ldaA/A-like at these stages. Consequently, cell bodies and proximal primary branches look white in the merged images, in addition to the overlapped branches that are marked by yellow arrows. Orange arrows (merge in G) point to spiracles. Genotype: (A–E) *UAS-mCherryCAAX, UAS-mmRFP/+; C161-Gal4, ppk-EGFP/ppk-EGFP* and (F and G) *ppk-CD4::tdTom/ppk-CD4::tdTom; C161-Gal4, UAS-mCD8::GFP/+*. Scale bars, 50 μm.

We previously imaged dendrites by using pan-neuron or pan-da neuron markers and found that the ldaA/A-like arbor is intermeshed with that of v'ada in the adult (right in Fig. 1; Shimono *et al*. 2009). To address how the two neuronal types develop individual dendritic arbors and whether dendritic branches of the two interact with each other, we differentially labeled them (Fig. 3).

**Figure 4:**
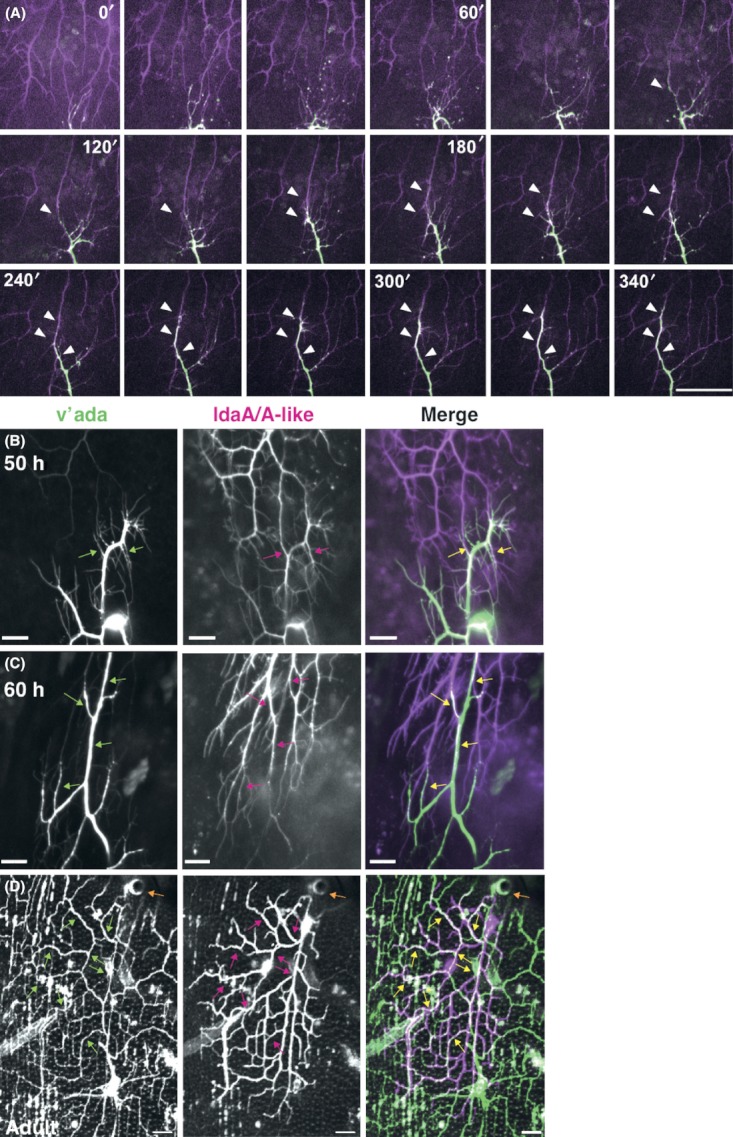
Dendrite–dendrite contacts of v'ada and ldaA/A-like at pupal and adult stages. (A) Time-lapse images of dorsally orienting dendrites of v'ada (green) and ventrally orienting ldaA/A-like dendrites (magenta) at 20-min intervals, from 48 h APF onwards. White arrowheads show the overlapped segments of v'ada and ldaA/A-like dendritic branches. See also Movie S6 (Supporting Information). (B–D) High-power images of the boxed regions in Fig. 3C,E,G, respectively. Dorsal dendrites of v'ada (left, green in the merged image at right) and ventral dendrites of ldaA/A-like (middle, magenta at right). Green, red and yellow arrows mark the overlap of filopodia and branches between v'ada and ldaA/A-like. In the middle panels of ‘B’ and ‘C’, note that the cell body (B) and proximal dendrites (B and C) of v'ada were labeled more weakly than ldaA/A-like. Orange arrow in ‘D’ points to a spiracle. Genotype: (A) *477-Gal4/UAS-mCherryCAAX, UAS-mmRFP; ppk-EGFP/ppk-EGFP* (B and C) *UAS-mCherryCAAX, UAS-mmRFP/+; C161-Gal4, ppk-EGFP/ppk-EGFP*, and (D) *ppk-CD4::tdTom/ppk-CD4::tdTom; C161-Gal4, UAS-mCD8::GFP/+*. Scale bars, 25 μm.

ldaA/A-like expanded its arbor ventrally in a unidirectional fashion, whereas v'ada developed a more radial arbor (Fig. 3A–D). Ventrally directing branches of ldaA/A-like encountered dorsal ones of v'ada approximately 50 h APF and they partially fasciculated with each other, which was seen at later stages as well (yellow arrows in Fig. 3C–G; high-power images are shown later in Fig. 4). At 60 h APF, ldaA/A-like arbors were more elaborated and larger than those of v'ada, and branches of these two neuronal types looked tightly associated with each other (Fig. 3E). The differences in arbor size and complexity of the two cell types were reversed after 72 h APF, making v'ada more expansive than ldaA/A-like (Fig. 3F). These spatiotemporal profiles of the dendritic development raised the possibility that ldaA/A-like, starting its arbor elaboration first, may serve as a guidepost to facilitate dorsal extension of later-growing v'ada branches, and that this dendrite–dendrite action eventually contributes to the establishment of adult dendritic arbors. This speculation prompted us to observe the dendrite–dendrite contact in more detail.

Time-lapse recordings showed that at least some of the filopodia sprouting from leading branches of v'ada and ldaA/A-like degraded upon initial contacts (Fig. 4A and Movie S6 in Supporting Information). Nonetheless, branches of both neurons started contacting with each other, and the overlapping region extended over time (white arrowheads in Fig. 4A). Thus, it is unlikely that the degeneration in this context elicits mutual repulsion of the branches. High-power images of the regions at 50 and 60 h APF showed that the overlap occurred not only between the branches, but also between filopodia and branches, and between filopodia of the different neuronal types (Fig. 4B,C). In 1-day-old adults, the v'ada arbor exhibits prominent radial-to-lattice transformation (Shimono *et al*. 2009; Yasunaga *et al*. 2010); the fasciculation was seen both in dorsoventral (DV) segments of the branches, which are embedded between muscles, and in anterior–posterior (AP) segments as well (Fig. 4D).

Roles of the dendrite–dendrite interaction have been shown *in vivo* in the context of avoidance between homotypic cells and self-avoidance of isoneuronal branches (Grueber & Sagasti 2010; Jan & Jan 2010; Matsubara *et al*. 2011; Lefebvre *et al*. 2012). In contrast, the heterotypic contact we observed implied its role as a guiding scaffold, as seen in many instances of local and/or transient cell–cell interactions in organizing neural circuit formation (Chao *et al*. 2009; Grueber & Sagasti 2010; Jan & Jan 2010).

### Attempts to selectively ablate ldaA/A-like by toxic gene expression

To address the above hypothesis, we used a system to eliminate either ldaA/A-like or v'ada selectively by expressing cell-death-inducing genes before the contact took place. We found that the expression of a dominant negative Rab5, DRab5[S43N] (Entchev *et al*. 2000), efficiently killed ldaA/A-like when driven by *C161-Gal4* (Shepherd & Smith 1996), and arbor formation of v'ada looked impaired in those pupae (results not shown). However, this *Gal4* system also induced gene expression in v'ada, and although the expression was weaker than that in ldaA/A-like (see details in the Fig. 3 legend), it complicated the interpretation of the results. In an attempt at shutting off the leaky expression in v'ada, we combined *C161-Gal4* with *ppk-Gal80* (Yang *et al*. 2009). Unfortunately, however, *ppk-Gal80* repressed Gal4 activity in ldaA/A-like as well as v'ada and could not be used for ldaA/A-like selective elimination (results not shown).

Therefore, we searched for *Gal4* stocks that could induce gene expression only in ldaA/A-like before or from the onset of the v'ada-ldaA/A-like contact. Of 156 lines tested, including a collection of taste-receptor gene drivers (Weiss *et al*. 2011), we isolated seven new *Gal4* lines that were specific to ldaA/A-like (Fig. S1 and Table S1 in Supporting Information). Unfortunately, however, none of them was early or strong enough for our purpose.

### Laser ablation of ldaA/A-like produces only marginal effects on the v'ada arbor

**Figure 5:**
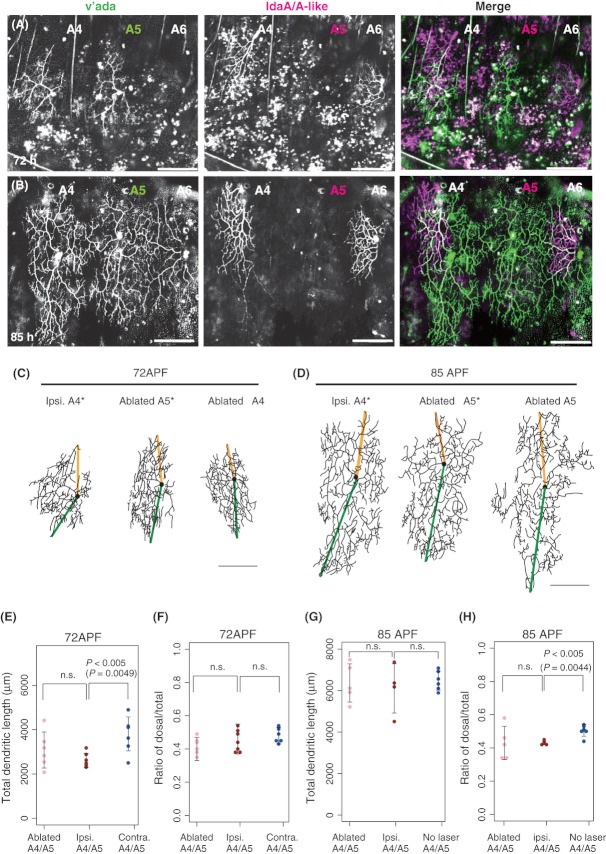
Ablation of ldaA/A-like produced only a marginal effect on morphogenesis of the v'ada arbor. (A and B) ldaA/A-like neurons of abdominal segment 4 or 5 (A4 or A5) were ablated at 40–42 h APF, and v'ada (left, green in right image) and ldaA/A-like (middle, magenta in right image) were imaged at 72 h (A) or 85 h (B) APF. The ablation was confirmed by the absence of ldaA/A-like neurons targeted in either A4 or A5. Genotype: *ppk-CD4::tdTom/ppk-CD4::tdTom; C161-Gal4, UAS-mCD8::GFP/+*. (C and D) Tracings of the dendrite arbors of v'ada at 72 h (C) and 85 h (D) APF. ‘Ipsi. A4’ indicates the v'ada arbor in A4 in a pupa where ldaA/A-like of the ipsilateral A5 was ablated. ‘Ablated A4’ indicates the v'ada arbor in A4 where ldaA/A-like in the same A4 was ablated. Asterisks indicate tracings of the v'ada neurons that are imaged in ‘A’ and ‘B’. Cell bodies and the most dorsal or ventral tips of branches are connected with yellow or green lines and used for calculating ratios of yellow/yellow + green in ‘F'; and ‘H’. Scale bars, 100 μm. (E–H) Quantitative analyses of dendritic arbors. (E and G) Total dendrite length of each v'ada arbor. ‘Ablated A4/A5’ and ‘Ipsi. A4/A5’ are as described above. ‘Contra. A4/A5’ indicates v'ada in A4/A5 segments contralateral to those where ldaA/A-like neurons were ablated. ‘No laser A4/A5’ indicates v'ada of pupae that were mounted as in the ablation protocol, but not irradiated. The data were statistically analyzed by a Welch's *t*-test. n.s., no statistically significant difference. (F and H) Ratio of length of the yellow line to the length of the yellow line plus the green line of each v'ada arbor. The data were analyzed by a Student's *t*-test. Each line with double blunt arrowheads is the mean ± SD.

Instead of genetically eliminating the neurons, we ablated ldaA/A-like using a laser and examined how the arbor size of v'ada was affected (Fig. 5). Because the origin of ldaA-like is not known (Shimono *et al*. 2009), the earliest stage when we were able to target both ldaA and ldaA-like was 40–42 h APF before the contact. We ablated ldaA/A-like in abdominal hemi-segment 4 or 5 (A4 or A5) and then imaged v'ada arbors at 72 or 85 h APF in the irradiated hemi-segments and in control hemi-segments (Fig. 5A–D). For each arbor, we quantified total branch length and the growth index along the DV axis (Fig. 5E–H) and assessed the effect of the ablation of ldaA/A-like on the v'ada arbor growth.

We had expected poor growth of at least dorsal branches of v'ada in the absence of ldaA/A-like in the same hemi-segments. However, visual observation did not show such gross morphological defects (Fig. 5A,B). Consistently, our quantitative analysis at the two time-points showed that the ablation of ldaA/A-like affected both parameters of v'ada arbors only marginally (Fig. 5E–H), and it was difficult to conclude that the ablation caused any significant differences. We also performed a converse ablation experiment, where v'ada was ablated either in wandering larvae or at 40–42 h APF and imaged ldaA/A-like at 72 h and 96 h APF. Again, we did not find obvious morphological defects in shaping arbors of the remaining neurons (Fig. S2 in Supporting Information; *n* = 5). Although we could not exclude the possibility that branches of the ablated neurons were invisible but somehow remained partially intact long after the ablation, our results under the conditions used showed that elongation and branching of dendrites did not appear to slow down in the absence of the heterotypic dendrite–dendrite contact and that the shape and size of the dendritic arbor in the regeneration phase could be specified almost normally. It could still be the case that the heterotypic contact might contribute in a longer term to restricting overshooting of branches or maintaining their arbors during adult life.

### Other possible mechanisms that shape dendritic arbors other than the dendrite–dendrite contact

It has been shown that the extracellular matrix (ECM) secreted by the epidermis makes significant contributions to shaping dendritic arbors of da neurons. In larvae, dendrite–substrate interactions ensure preventing crossings of isoneuronal branched or self-avoidance (Han *et al*. 2012; Kim *et al*. 2012), whereas local degradation of the ECM in newly eclosed adults plays a pivotal role in reshaping radial arbors of v'ada into the lattice shape (Yasunaga *et al*. 2010). It could be that branch elongation of v'ada and ldaA/A-like neurons may be particularly promoted in a spatially restricted fashion by an unknown ECM distribution between the cell bodies of the two cell types, which secondarily results in the dendrite–dendrite contact. How the arbor growth is restricted at the end of pupal development remains to be elucidated. The expansive arbor of v'ada occupies the entire pleura (the lateral plate of the adult abdomen), but its dorsal and ventral borders do not necessarily about those of ddaE neuron in the tergite and v'ada in the contra-lateral hemi-segment, respectively (see fig. 3 in Shimono *et al*. 2009). Given that the pupal dendrites cope with spatial and hormonal environments distinct from those in larvae, we should be able to attain novel mechanistic insights into shaping dendritic arbors with the v'ada arbor as an assay system and the *in vivo* time-lapse imaging developed in this study.

## Experimental procedures

### Fly stocks

We used the Gal4-UAS system (Brand & Perrimon 1993) to express most of the transgenes and to visualize da neurons. Gal4 driver strains used were *C161Gal4* (Shepherd & Smith 1996), *Gal4*^*109(2)80*^ (Gao *et al*. 1999), *477-Gal4* (Grueber *et al*. 2003), *Gr-Gal4* lines (Weiss *et al*. 2011; kindly provided by Dr Carlson) and those in Table S1 (Supporting Information) (the Bloomington stock center). UAS stocks were *UAS-GFP[S65T]*, *UAS-mCD8:GFP* and *UAS-mmRFP* (the Bloomington Stock Center), *UAS-mCherryCAAX* (Kakihara *et al*. 2008) and *UAS-DRab5[S43N]* (Entchev *et al*. 2000). Other stocks were *ppk-EGFP* (Grueber *et al*. 2003), *ppk-CD4::tdTom[4a]* (Han *et al*. 2011) and *ppk-Gal80* (Yang *et al*. 2009). All pupae and adults were grown at 25 °C.

### Image acquisition of whole-mount animals and quantitative analysis

To acquire images of da neurons at pupal stages, the collected pupae were transferred to a plate filled with water, washed and dried on 3MM Chr paper (Whatman). Each pupa was taken out of its puparium carefully by forceps, mounted in PBS on a slide between spacers made of vinyl tape, and covered with a 24 × 24 mm cover slip (No. 1, MATSUNAMI). For imaging, adult abdomens, heads, wings and legs were removed and mounted in 50% glycerol as described above. Images of ldaA/A-like and v'ada neurons at A4 and/or A5 were acquired by laser scanning confocal microscopy (NikonC1) with 488- or 543-nm lasers with a 1-μm Z-step and total 15–20 μm depth. Images were processed by using Photoshop, Illustrator (Adobe Systems), and ImageJ. Neurocyte software (Kurabo) was used for the quantification of total dendritic length. Statistical analysis was performed by R program (version 2.14.0; The R Foundation for Statistical Computing).

### Time-lapse recording

Each pupa was taken out of its puparium as described above and mounted on a 35-mm glass-bottomed dish (3911-035, IWAKI). In mounting, we folded legs and put abdomens on the dish and tilted them to retain an appropriate angle to observe v'ada and/or ldaA/A-like neurons. Details of image acquisition are essentially described previously (Harumoto *et al*. 2010), except for Movie S1 (Supporting Information). Briefly, Movies S2–S6 (Supporting Information) were acquired by using a spinning-disk confocal scan head (CSU10; Yokogawa), an Olympus IX71 microscope and an EM-CCD camera (DU-888; Andor Technology). Fluorescent proteins were excited with a 488-nm line and a 561-nm line, and signals were detected with a 500- to 550-nm and 580- to 640-nm band-pass filter, respectively. The exposure time was 500 ms for both 488 and 561 nm, and the EM gain of the camera was 1000×. The output of the 488- and 561-nm laser power was 10% and 40%–50%, respectively. Typically, single confocal planes were taken with a 0.7-μm Z-step and total 15–20 μm depth, at 30-s or 60-s intervals. The above hardware was driven by MetaMorph (Molecular Device), and acquired data were processed with MetaMorph and ImageJ. After image acquisition, each pupa was kept at 25 °C and their survival was confirmed to at least the pharate stage or the adult stage. Movie S1 (Supporting Information) was acquired by using a Leica TCS SPE (see its legend).

### Ablation experiment

In our ablation experiments, pupae were mounted as described in ‘Image acquisition of whole-mount animals and quantitative analysis’, except water was substituted for PBS. Both cell bodies and trunks of dendrites of ldaA/A-like were targeted at 40–42 h APF through a 100× UPlanApo objective that was attached to a microscope (BX51; OLYMPUS), and ablated by using a 337-nm N2 laser at a frequency of 20 Hz for 5–10 s (Micropoint, Photonics Instruments). This condition was harsher than what was used for ablating da neurons in embryos and larvae (Sugimura *et al*. 2003). Fluorescence of cell bodies was no longer detected after 1 min. The pupae were kept at 25 °C until 72 or 85 h APF. To collect six hemi-segments at 72–74 h APF where both ldaA and ldaA-like were ablated, we targeted 19 hemi-segments at 40–42 h APF (Fig. 5E,F). Similarly, we targeted 12 hemi-segments at 40–42 h APF to collect five hemi-segments at 85–87 h APF where the ablation was successful (Fig. 5G,H).

## Supporting Information/Supplementary Material

**Figure S1** ldaA/A-like-specific Gal4 drivers.

**Figure S2** ldaA/lda-A like developed arbors in the absence of v'ada branches.

**Table S1** ldaA/A-like specific Gal4 drivers in pupal and adult stages

**Movie S1** The ventral region of the abdominal hemisegment at 14 h APF onwards

**Movie S2** Dynamic filopodia extended from a primary branch of v'ada at 40–46 h APF

**Movie S3** Local degeneration of filopodia of v'ada at 55 h APF

**Movie S4** Growth of thick primary branches at 60–64 h APF

**Movie S5** Conversions of dynamic filopodia into stable branches at 64–68.5 h APF

**Movie S6** Ventral growing dendrites of ldaA/A-like were overlapped with dorsal dendrites of v'ada at 48–56 h APF
