# Supplementary material for: High-resolution in vivo imaging of regenerating dendrites of Drosophila sensory neurons during metamorphosis: local filopodial degeneration and heterotypic dendrite–dendrite contacts
Source: Genes Cells. 2012 Nov 15;17(12):939–51. doi: 10.1111/gtc.12008 (PMC3549480; doi:10.1111/gtc.12008)
Supplement: Supplementary file 1 [file gtc0017-0939-SD7.docx]

**
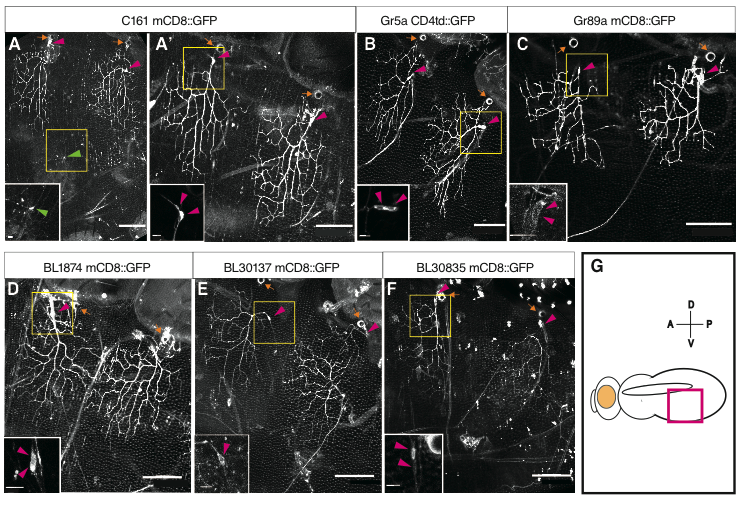
**

**Figure S1. ldaA/A-like-specific Gal4 drivers**

(A-F) Six Gal4 drivers that specifically labeled ldaA/A-like at the adult stage. Pleura (lateral plate) of two abdominal hemisegments (A4 and A5) were imaged and yellow boxed regions are magnified in the insets. Green and magenta arrowheads mark cell bodies of v’ada and ldaA/A-like neurons, respectively. Orange arrows indicate spiracles. Genotype: (A and A’) *C161 UAS-mCD8:GFP/+*, (B) *Gr5a UAS-CD4td::GFP/Gr5a UAS-CD4td::GFP*, (C) *Gr89a UAS-mCD8::GFP/+*, (D) *BL1874 UAS-mCD8::GFP/BL1874 UAS-mCD8::GFP*, (E) *BL30137 UAS-mCD8::GFP/+*, (F) *BL 30835 UAS-mCD8::GFP/BL 30835 UAS-mCD8::GFP*. Scale bars, 100 µm and 10 µm (insets). (G) Schematic lateral view of an adult. A magenta boxed region was imaged.

**
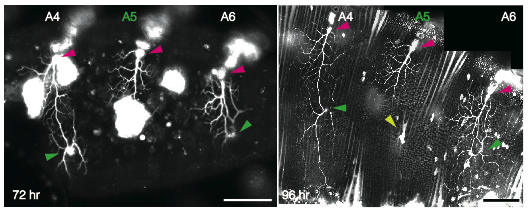
**

**Figure S2. ldaA/lda-A like developed arbors in the absence of v’ada branches**

v’ada of A5 was ablated at 40-42 hr APF and ldaA/A-like were imaged at 72 hr and 96 hr APF. Genotype: *Gal4^109(2)80^* *UAS-GFP[S65T]/ Gal4^109(2)80^* *UAS-GFP[S65T]*. Green and red arrowheads mark cell bodies of v’ada and ldaA/A-like neurons, respectively; and in A5, the irradiated v’ada may have survived but failed to extend branches (yellow arrowhead). Scale bars, 100 µm.

**
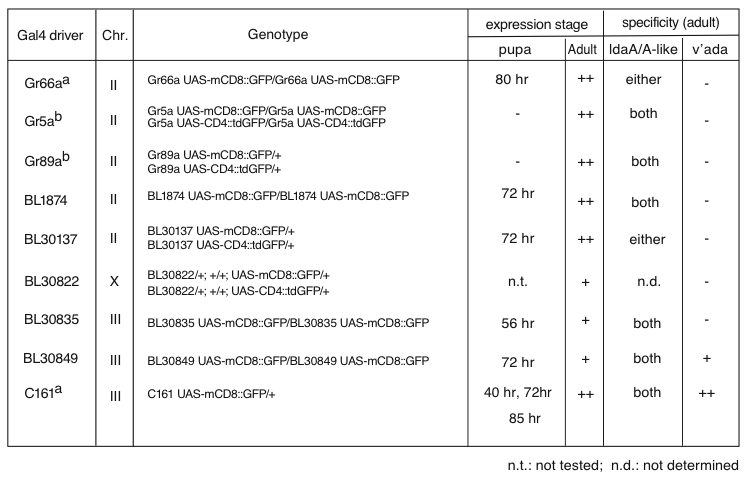
**

**Table S1. ldaA/A-like specific Gal4 drivers in pupal and adult stages**

Signals were imaged in whole-mount adults and their intensity was classified as follows: ++ (strong); + (variable intensity between segments); - (not detected); and n.d. (not determined): difficult to distinguish whether both of ldaA and ldaA-like were labeled or not. Pupal stages when signals were detected are specified. a: Dunipace *et al*, 2001 and b: Weiss *et al*, 2011. Other Gal4 drivers are from the Bloomington Stock Center. The data for Gr66a were previously reported (Shimono et al., 2009).

**Movie S1. The ventral region of the abdominal hemisegment at 14 hr APF onwards**

A time-lapse recording between 14 hr and 62 hr APF, taken with a 1.5 µm Z-step and at 10 min intervals. See the legend of Figure 1 for explanations. Genotype: *Gal4^109(2)80^* *UAS-mCD8::GFP*/*Gal4^109(2)80^* *UAS-mCD8::GFP*. Scale bar, 250 µm.

**Movie S2. Dynamic filopodia extended from a primary branch of v’ada at 40-46 hr APF**

In this and three subsequent movies, primary dendrites and cell bodies of v’ada were imaged with a 0.7 µm Z-step and at 30 sec intervals. The tip of the primary branch has a lamellipodium. Genotype: *ppk-EGFP/ppk-EGFP.* Scale bar, 20 µm.

**Movie S3. Local degeneration of filopodia of v'ada at 55 hr APF**

Selected frames are shown in Figure 2E’ and 2E”. Anterior is to the right and dorsal is up. Genotype: *ppk-EGFP/ppk-EGFP.* Scale bar, 20 µm.

**Movie S4. Growth of thick primary branches at 60-64 hr APF**

See also Figure 2F. Genotype: *ppk-EGFP/ppk-EGFP.* Scale bar, 20 µm.

**Movie S5. Conversions of dynamic filopodia into stable branches at 64-68.5 hr APF**

See also Figure 2F’. Genotype: *ppk-EGFP/ppk-EGFP.* Scale bar, 20 µm.

**Movie S6. Ventral growing dendrites of ldaA/A-like were overlapped with dorsal dendrites of v’ada at 48-56 hr APF**

v’ada (left, green in right) and ldaA/A-like neurons (middle, red in right) were imaged at 60-second intervals. See also Figure 4A. Genotype: *477-Gal4/UAS-mCherryCAAX, UAS-mmRFP; ppk-EGFP/ppk-EGFP*. Scale bar, 20 µm.

**References**

Dunipace, L., Meister, S., McNealy, C., & Amrein H. (2001) Spatially restricted expression of candidate taste receptors in the Drosophila gustatory system. *Current Biology*. **11**, 822-35.

Shimono, K., Fujimoto, A., Tsuyama, T., Yamamoto-Kochi, M., Sato, M., Hattori, Y., Sugimura, K., Usui, T., Kimura, K. & Uemura, T. (2009) Multidendritic sensory neurons in the adult Drosophila abdomen: origins, dendritic morphology, and segment- and age-dependent programmed cell death. *Neural Dev* **4**, 37.

Weiss, L.A., Dahanukar, A., Kwon, J.Y., Banerjee, D. & Carlson, J.R. (2011) The molecular and cellular basis of bitter taste in Drosophila. *Neuron* **69**, 258-272.
